# Depression and Eye Disease—A Narrative Review of Common Underlying Pathophysiological Mechanisms and their Potential Applications

**DOI:** 10.3390/jcm13113081

**Published:** 2024-05-24

**Authors:** Wymann Shao Wen Tang, Nicole Xer Min Lau, Muthuramalingam Naveen Krishnan, You Chuen Chin, Cyrus Su Hui Ho

**Affiliations:** 1 Yong Loo Lin School of Medicine, National University of Singapore, Singapore 119077, Singapore; 2 Raffles Medical Group, Singapore 188770, Singapore; 3 Department of Psychological Medicine, National University of Singapore, Singapore 119077, Singapore; 4 Department of Psychological Medicine, National University Hospital, Singapore 119228, Singapore

**Keywords:** depression, dry eye disease, cataracts, glaucoma, macular degeneration, diabetic retinopathy, eye disease, optical coherence tomography, consultation-liaison psychiatry

## Abstract

**Background:** Depression has been shown to be associated with eye diseases, including dry eye disease (DED), cataracts, glaucoma, age-related macular degeneration (AMD), and diabetic retinopathy (DR). This narrative review explores potential pathophysiological connections between depression and eye disease, as well as its potential correlations with ocular parameters. **Methods**: A literature search was conducted in August 2022 in PUBMED, EMBASE, and PsycINFO. Published articles related to the subject were consolidated and classified according to respective eye diseases and pathophysiological mechanisms. **Results**: The literature reviewed suggests that common pathophysiological states like inflammation and neurodegeneration may contribute to both depression and certain eye diseases, while somatic symptoms and altered physiology, such as disruptions in circadian rhythm due to eye diseases, can also influence patients’ mood states. Grounded in the shared embryological, anatomical, and physiological features between the eye and the brain, depression is also correlated to changes observed in non-invasive ophthalmological imaging modalities, such as changes in the retinal nerve fibre layer and retinal microvasculature. **Conclusions**: There is substantial evidence of a close association between depression and eye diseases. Understanding the underlying concepts can inform further research on treatment options and monitoring of depression based on ocular parameters.

## 1. Introduction

Depression is a prevalent comorbidity in patients with eye diseases, including dry eye disease (DED), glaucoma, age-related macular degeneration (AMD), cataract, and diabetic retinopathy (DR), amongst others [1]. Visual impairment resulting from these eye diseases can contribute to depression stemming from a diminished quality of life and impaired overall functioning. This is well supported by evidence from meta-analyses such as that conducted by Parravano et al., showing a higher prevalence of depression amongst 6992 patients with visual impairment across 27 studies [2]. However, depression is not an inevitable outcome of visual impairment, and it has also been shown that depression can occur at varying levels of visual impairment [3].

It has been suggested that, owing to their common origins in the neural tube, the retina may act as a window to the brain by displaying structural and functional changes that may assist in diagnosing psychiatric conditions such as depression and even neurodegenerative conditions [4,5]. Furthermore, the available literature points towards multiple promising biomarkers for depression, including inflammatory, endocrine, and oxidant stress markers, amongst others, suggesting probable systemic pathophysiology driving the symptomatology of depression that may also affect the eye [6,7,8]. This means that while depression may be a response to eye disease, the relationship between the two may also likely be bidirectional, driven by biological mechanisms with psychiatric and ophthalmological manifestations. Recognising and characterising these correlations would allow clinicians to detect cases for timely intervention and provide more well-rounded patient care. In time, this may provide objective measures for diagnosis and prognostication in clinical practice for identification of cases and treatment monitoring. 

However, more research is required to understand the link between eye diseases and depression. Despite numerous studies, research on their relationship and common mechanisms is lacking. This narrative review aims to examine data from existing studies on the relationship between depression and significant eye diseases while uncovering common underlying pathophysiologic mechanisms.

## 2. Methods

Three databases (PubMed, Embase, and PsycINFO) were last searched on 29 August 2022, with no restrictions on publication dates. A combination of keywords such as ‘depression’, ‘retinal microvascular changes’, ‘optical coherence tomography’, ‘retinal nerve fiber layer’, ‘diabetic retinopathy’, ‘macular degeneration’, ‘glaucoma’, ‘cataracts’, ‘dry eye disease’, and ‘ocular surface disease’ were employed in the search with the use of relevant controlled vocabulary such as Medical Subject Headings, Emtree, and PsycINFO Thesaurus terms. 

Two authors, W.S.W.T. and NLXM, selected the articles for this review using a two-phase process. In the first phase, they screened articles based on their title and abstracts to determine their relevance. Articles that passed the first screening phase were further evaluated through full-text assessments to determine their eligibility for inclusion in the review. The review included studies that provided evidence of the association, risk factors, and possible pathophysiological links between various eye diseases and depression or depressive symptoms. The studies included in the review were cross-sectional studies, cohort studies, case-control studies, randomised controlled trials, quasi-experimental studies, reviews, and meta-analyses. If there were any disagreements during the selection process, the authors discussed the issues and consulted with the senior author, CSHH, to resolve any conflicts. A repeated search was subsequently conducted on 14 May 2024 to supplement the review with recent articles published since the earlier database search. Selected articles were supplemented with hand-searching of relevant articles on the aforementioned databases for further corroboration and synthesising of information. A total of 136 articles were reviewed and assessed for the results section.

## 3. Association of Depression with Eye Diseases

### 3.1. Depression and DED

#### 3.1.1. Epidemiology

Meta-analyses have shown that DED is frequently associated with depression. Patients with DED have a higher prevalence of depression than those with other eye diseases, with an odds ratio of 2.92 [1,9,10,11]. The severity of DED symptoms is significantly linked to the severity of depressive symptoms [12]. However, there is no significant correlation between depression severity and signs of DED, such as tear break-up time (TBUT), Schirmer’s test, and corneal fluorescein staining [10].

#### 3.1.2. Mechanisms

##### Inflammation

In DED, inflammation affecting the eye surface leads to blinking, tearing, and corneal sensation disruptions [13]. Primary Sjogren’s disease, an autoimmune disorder, correlates with a higher incidence and severity of depression, suggesting inflammation is a critical link between depression and DED in chronic inflammatory diseases [9]. Patients with depressive symptoms in DED show increased pro-inflammatory receptor and cytokine expression, with elevated P2X7 receptor in Sjogren’s patients correlating with higher IL-1B and depression scores [14,15]. Studies reveal elevated IL-17 and TNF-alpha in tear fluid of depressive DED patients, correlating with DED severity [14]. Other autoimmune diseases like ankylosing spondylitis, rheumatoid arthritis, and systemic lupus erythematosus are associated with depressive symptoms, indicating shared inflammatory pathways [16].

##### Dysregulation of Sleep

Studies indicate a significant correlation between DED, depression, and sleep disorders. Cross-sectional investigations have identified a psychiatric symptom complex, including depression and sleep disorders, associated with DED [17,18,19,20]. Notably, reduced sleep duration and diminished sleep quality, measured by the Pittsburgh Sleep Quality Index (PSQI), are prevalent among DED patients. Researchers have established a link between sleep quality and the severity of both DED and depression scores [18,19]. Moreover, the severity of depression in DED patients is influenced by subjective sleep quality and sleep latency [21]. A bidirectional relationship is proposed, where DED symptoms may induce depression and sleep disorders, and, conversely, sleep disturbances may result from DED-related factors, leading to depression [20]. Consequently, recognising and addressing sleep disturbances are crucial in managing comorbid depression in individuals with DED.

##### Antidepressants

Selective serotonin reuptake inhibitors (SSRIs) may exacerbate depression in DED patients through the pro-inflammatory NF-KB pathway [22]. Individuals with both DED and depression on SSRIs exhibit elevated tear serotonin levels and ocular inflammation. Human corneal epithelial cell studies support serotonin’s role in inflammation through NF-KB signalling [22]. Studies by Isik-Ulusoy et al. and Kocer et al. showed that SSRIs and serotonin and norepinephrine reuptake inhibitors (SNRIs) can heighten objective and subjective parameters of DED, complicating the understanding of the depression–DED association, as antidepressant use may not be consistently considered in studies [23,24]. Depression and antidepressant use are independently associated with DED [25]. In addition to sharing a common pathophysiology, pharmacological interventions for depression contribute to this increased association with DED, impacting patient management.

##### Genetic Linkages

In addition to providing further evidence in confirming the bidirectional nature between DED and depression via meta-analysis and Mendelian randomisation, the multi-modal study by Chang et al. further established probable genetic associations between the two disease entities through genetic wide-associations studies based on data from the East Asian and UK biobanks [11]. Variant-disease association (VDA) studies and disease–disease association (DDA) studies reveal commonalities in genetic architecture (such as MUC16 and TENM2 mutations) contributing to the development of both diseases [11].

### 3.2. Depression and Cataracts

#### 3.2.1. Epidemiology

The pooled prevalence of depression and depressive symptoms amongst cataract patients is as high as 25% [1]. Poor high-contrast vision and quality of life, as well as higher self-reported visual disability and comorbidity scores, predicted depressive symptoms in these patients [26]. Cataracts are associated with an increased risk of depression, but surgery can reduce this risk and improve cognitive function [27,28]. Improved vision after surgery is linked with better depression scores [29,30].

#### 3.2.2. Mechanisms

##### Dysregulation of Sleep

Cataracts increase depression risk by affecting vision and disrupting sleep. Age-related lens changes reduce the transmission of short-wave light, impacting the circadian rhythm and causing insomnia and depression [31,32,33]. Studies found that blue-blocking intraocular lenses (IOLs) increased depression risk, while patients with clear IOLs had lower rates of depression [34,35]. Of note, Lereuz et al.’s study found no significant difference in the improvement of depression scores between patients fitted with blue light filtering IOLs and those provided with conventional IOLs [36]. Nonetheless, enhanced vision post cataract surgery may outweigh the blue light-blocking lens impact on depression risk. These findings emphasise light exposure’s relevance in depressive symptoms influenced by cataracts.

##### Dysregulation of the Hypothalamic-Pituitary-Adrenal (HPA) Axis 

Cataract formation is a recognised complication of Cushing’s syndrome, particularly when triggered by exogenous steroids. Exposure to glucocorticoids is believed to induce gene expression changes and activate receptors in lens epithelial cells, contributing to cataract development [37]. Additionally, systemic complications arising from hypercortisolism, such as type 2 diabetes mellitus (T2DM), hypertension, and metabolic syndrome, significantly elevate the risk of cataracts [38]. In depression, evidence reveals dysregulation of the HPA axis, with increased plasma cortisol associated with severe depression, particularly with melancholic features stemming from stress-related cortisol release [39]. Elevated HPA activity, linked to impaired negative feedback mechanisms, responds positively to antidepressant treatment through glucocorticoid receptor upregulation [40]. These HPA axis changes are also associated with impaired cognitive function, treatment resistance, and frequent relapses in depression [41,42].

### 3.3. Depression and Glaucoma

#### 3.3.1. Epidemiology

Amongst glaucomatous patients, duration of glaucoma, visual function, and quality of life (QoL) score predict depression [43,44,45]. Risk factors for depression include older age, increased glaucoma severity, and burden of comorbidity [46,47]. Primary angle closure glaucoma patients may experience more severe depressive symptoms [48]. Depressed patients have a higher risk of developing glaucoma, while the presence of anxiety or depression in individuals identified as glaucoma suspects were more likely to develop glaucoma during the follow-up period [49].

#### 3.3.2. Mechanisms

##### Inflammation

While not fully integrated into clinical practice, research indicates the involvement of neuro-inflammation and para-inflammation dysfunction in areas like the retinal ganglion cell layer (GCL), optic nerve head, trabecular meshwork, and ocular surface in glaucoma [50]. This aligns with the concept of inflammation as a shared factor in depression and glaucoma.

##### Dysregulation of Sleep

Studies show a connection between subjective sleep quality measured by the PSQI and visual field loss, along with depressive symptoms in glaucoma patients [51,52,53]. Agorastos and Huber’s review highlighted potential mechanisms, emphasising the role of melatonin. Melatonin secretion relies on intrinsically photosensitive retinal ganglion cells (ipRGC), which are progressively lost in glaucoma [54]. This is supported by findings in patients with primary open-angle glaucoma that RGC loss measured by optical coherence tomography (OCT) is strongly associated with depressive symptoms [55].

##### Medications for Glaucoma

Eye medications may cause depression, but the findings are inconsistent. A study found that using topical timolol for glaucoma increased the risk of depression, while another study found no effect [56,57]. The reasons and mechanisms for this probable relationship are still unclear.

##### Antidepressants

Antidepressants, especially SSRIs, can increase the risk of glaucoma depending on the duration and dosage of treatment [58,59]. Another study showed that starting SSRIs may be linked to acute angle closure glaucoma [60]. SSRIs can affect intraocular pressure balance by stimulating various eye serotonin receptors in the ciliary complex, epithelial cell layer, and pupillary sphincter, increasing aqueous humour production and causing mydriasis. This can increase outflow tract obstruction [61,62].

### 3.4. Depression and AMD

#### 3.4.1. Epidemiology

Depression is prevalent in patients with AMD, with a cross-sectional study revealing a 32.5% rate among older adults with advanced AMD, twice the general population’s 10.5% [63,64]. Observational and longitudinal studies, including the Canadian Longitudinal Study on Ageing and a study based on the Korean National Health Screening Program, confirm AMD as a risk factor for incident depression in the elderly [65,66,67,68]. Risk factors for depression in patients with AMD include poor self-perception of health, impaired activities of daily living (ADLs), and poor visual function, while higher perceived stress correlates with reduced visual function in neovascular AMD [69].

#### 3.4.2. Mechanisms

##### Inflammation

Fluoxetine, an SSRI used for depression, effectively inhibits NLRP3-ASC inflammasome activation and the release of inflammatory cytokines in retinal pigment epithelium (RPE) cells and macrophages, potentially mitigating dry AMD [70]. Depression patients treated with fluoxetine show a reduced risk of developing dry AMD, indicating a shared therapeutic effect. The NLRP3 inflammasome, implicated in depression risk through stress-triggered proinflammatory cytokines, also drives depression development via pyroptosis [71]. In animal models, blocking NLRP3 activation reduces depressive-like behaviours. This suggests a potential link between depression and AMD through shared inflammatory processes [72,73,74,75].

##### Dysregulation of Sleep

ipRGCs transmit signals to the brain that regulate sleep–wake cycles, sleep quality, and mood. These parts of the brain include the suprachiasmatic nucleus and ventrolateral pre-optic area [76,77,78,79,80]. A study by Maynard et al. measured ipRGC function using melanopsin-mediated post-illumination pupil response (PIPR). It examined subjects with and without AMD for correlation with sleep efficiency, quality, and depression. The group with AMD had poorer ipRGC function, lower global sleep scores, and higher depression levels. However, the ipRGC function only correlated with sleep efficiency and quality, not depression levels [81]. However, this study could not clarify if AMD affects ipRGCs’ projection to mood centres [81]. Nevertheless, depressive symptoms are linked to poor sleep quality. Better sleep improves mental health [82,83].

##### Dysfunction in Brain Neural Homogeneity 

Liu et al. investigated cerebral homogeneity in AMD patients using the regional homogeneity (ReHo) method, finding dysfunction in brain neural homogeneity [84]. This may be a mechanism for chronic vision loss, anxiety, and depression in AMD. ReHo data could serve as an early screening tool for AMD. In AMD patients, limbic lobe and parahippocampal gyrus activity increased, while cingulate and superior frontal gyrus activity decreased. Cingulate and superior frontal gyrus activity inversely correlated with depression scores [84]. Previous studies support these findings, linking the cingulate gyrus to depression development and emphasising deep brain stimulation in improving treatment-resistant depression [85,86]. Abnormalities in the prefrontal cortex, specifically the superior frontal gyrus, consistently increase depression vulnerability. Liu et al.‘s study provides direct evidence of altered brain activity affecting depression pathways in AMD [84]. However, further research is needed to confirm this association and assess the method’s effectiveness in gauging depression risk in AMD patients.

##### Medications for AMD

Senra H’s study suggests that early stages of anti- vascular endothelial growth factor (anti-VEGF) treatment for AMD may contribute to depressive symptoms [87]. Qualitative data on treatment experiences revealed clinical depression in 12% of patients. Significantly higher rates were observed in those receiving up to 3 injections than those receiving 4–12 injections (Analysis of Variance [ANOVA]) *p* = 0.027) or more than 12 injections (ANOVA *p* = 0.001). The phenomenon is believed to be caused by the stress associated with intravitreal injections during the initial stages of treatment. Situations causing delays in injections may also contribute to stress in AMD patients [88]. However, it may also possibly be attributed to transient increases in intra-ocular pressures (IOP) post-injection [89]. Nonetheless, the study does not account for different types of intra-vitreal injections–for instance, evidence suggests IOP post-injection is higher for bevacizumab and ranibizumab than aflibercept [90]. The correlation between IOP and depressive symptoms should be further evaluated and reconciled with the benefits of anti-VEGF injection on quality of life by improving or maintaining visual function [91]. 

##### Antidepressants

Mantel et al.’s study indicates that antidepressant medications, including SSRIs and SNRIs, may increase anti-VEGF requirements in neovascular AMD patients [92]. This prospective study revealed significantly more pigment epithelium detachment in AMD patients on antidepressants [92]. This observation parallels findings in DED, suggesting that the association between AMD and depression may be influenced by antidepressant use in individuals with baseline depression. The discrepancy in the impact of antidepressants on AMD, compared to Ambati et al.’s findings, may be attributed to the distinct AMD types they studied [70]. Mantel et al. focused on neovascular AMD, characterised by abnormal vessel formation in the subretinal space [92]. In contrast, Ambati et al. concentrated on dry AMD, marked by retinal pigment epithelium degeneration and atrophy [70]. Mantel et al.’s results align with prior research demonstrating that antidepressants, particularly SNRIs, induce VEGF, acting as a mediator for their behavioural effects [93]. Elevated markers of VEGF in patients with depressive disorders, including gene polymorphisms, VEGF mRNA expression, and plasma VEGF levels, suggest a neuroprotective role for VEGF in response to clinical depressive states [94,95,96]. Consequently, the collective evidence suggests that heightened VEGF, triggered by both depressive states and antidepressant medications, may contribute to the development of neovascular AMD.

### 3.5. Depression and DR

#### 3.5.1. Epidemiology

Several meta-analyses of cross-sectional and cohort studies have shown that depression is significantly associated with DR in patients with DM [97,98]. Conversely, evidence from cross-sectional studies suggests that the severity of both proliferative and non-proliferative DR is associated with the incidence and severity of depressive symptoms [99,100,101]. Specifically, patients with DR and higher depressive scores have been shown to have poorer diabetes self-management [102]. 

#### 3.5.2. Mechanisms

##### Inflammation

Systemic inflammation may be a common cause of depression, DM, and DR. Older diabetic individuals with mild cognitive impairment may experience depressive symptoms if there are high levels of C-reactive protein (CRP) and adhesion molecules in their blood. The connection between inflammation, depression, and DR is not yet fully understood [103].

##### Dysregulation of the HPA Axis 

Chronic hyperinsulinaemia in patients with T2DM leads to HPA axis hyperactivity, contributing to metabolic syndrome [104,105,106]. This syndrome includes hyperglycaemia, dyslipidaemia, and hypertension—significant risk factors for DR [107]. Hyperglycaemia, worsened by hypercortisolism, is implicated in metabolic pathways key to the development of DR, such as the polyol pathway and advanced glycation end-product formation [108,109].

##### Systemic Effects of Diabetes Mellitus

Depression and DR may be linked to poor DM management due to stress and the other health complications that come with the condition [110]. Risk factors such as obesity, lack of exercise, and chronic inflammation increase the likelihood of both depression and DM [110]. Furthermore, DM is a significant cardiovascular risk factor and supports the idea that there is a strong connection between vascular pathology and depression [111,112].

##### Antidepressants

Depression’s link to DR is not fully understood. According to Yekta et al., SSRIs may lower DR risk. However, the link’s mechanisms are unclear, and it does not necessarily suggest a shared pathophysiology [113].

##### Neurodegeneration

Retinal neurodegeneration is an independent pathophysiological mechanism in the progression of DR, evident in both animal and human models [108,114,115]. Brain-derived neurotrophic factor (BDNF), known for its neuroprotective role in the retina, is proposed to optimise retinal health [116]. Additionally, BDNF, implicated in neuroplasticity, has shown promise as a serum biomarker for depression, where decreased levels may predispose individuals to depression [6,7,8]. Supporting the link between depression and DR via BDNF, a cross-sectional study revealed a negative correlation between BDNF levels and depressive symptoms in diabetic patients [117]. A cohort study further indicated that low BDNF was associated with an increased risk of diabetic complications [118]. These findings suggest that the shared mechanism of neurodegeneration and reduced neuroplasticity may underlie the connection between depression and DR.

## 4. Depression and Retinal Microvascular Changes

### 4.1. Physiological Basis

The retina and brain share similar embryological origin and physiology, making the retina useful in studying microvascular changes [119]. The vascular depression hypothesis, which posits that cerebrovascular disease and its contributing risk factors may contribute to late-life depression symptoms, also suggests that retinal microvascular changes may be a potentially helpful tool in predicting the risk of depression [120]. Depression has been associated with micro- and macrovascular disease, including total white matter hyperintensity volumes on magnetic resonance imaging (MRI) and retinopathy [121]. Chronic depression symptoms could lead to vascular dysfunction through nitric oxide regulation alterations [122].

### 4.2. Parameters

Research indicates a connection between depression and retinal microvascular changes, including retinopathy, alterations in vessel calibre, and vessel occlusion, mirroring the relationship observed in diabetes-related microvascular complications [123]. Depression correlates with increased retinal venular calibre, arteriolar narrowing, and hypoperfusion [122]. Amongst eye diseases causing visual impairment, retinal vein occlusion is noted to have one of the highest prevalences of depression [124]. Retinal vein occlusion is linked to changes in the microvasculature associated with depression. In individuals with T2DM and depression, wider arteriolar width in the retina has been observed [125]. Table 1 highlights associations between a wider retinal venular or arteriolar calibre, reduced arterial tortuosity, and higher plasma markers of endothelial dysfunction with an increased prevalence or incidence of depression [122,126,127,128,129]. However, inconsistencies in reporting this association exist, potentially attributable to selection bias, variations in retinal microvascular changes, and differences in measurement time points [130,131].

### 4.3. Depression and Optical Coherence Tomography (OCT) Parameters

#### 4.3.1. Physiological Basis

Dysregulation of inflammatory cytokines may contribute to the development of depression through neurodegeneration and neuroinflammation [132]. Depression is also prevalent as a prodromal symptom of neurodegenerative disorders such as dementia [133]. Studies have found that thinning of the retina nerve fibre layer (RNFL) and ganglion cell layer (GCL) were associated with poor white-matter microstructure, while inner plexiform layer (IPL) thinning was linked to grey matter loss in the temporal and occipital lobes [134,135]. In multiple sclerosis, an inflammatory neurodegenerative disease that causes demyelination and axonal loss resulting in neurological deficits, strong evidence was also found concerning its association with RNFL and GC-IPL thinning and atrophy [136]. The retina is an ideal proxy for understanding neurodegenerative processes related to depression because of the similarities in anatomical and physiological characteristics between the central nervous system and retina and their connection via the optic nerve [137].

#### 4.3.2. Parameters 

OCT studies reveal a significant association between thinning in the RNFL and depression, with GCL and IPL thickness also reduced in patients with depression, correlating with duration and family psychiatric history [67,138,139,140,141]. However, conflicting findings, including increased RNFL thickness, may stem from sample size, medical comorbidities, selection bias, and measurement of OCT parameters in both eyes [141,142,143,144,145]. Neurodegeneration and inflammation may initially induce hypervascularity and increased choroidal thickness, followed by degeneration and thinning with repeated depressive episodes [140,141,145]. Thinning rates vary across retinal regions, with GCL and IPL affected before peripapillary RNFL [145]. These mechanisms may explain discrepancies in study findings. RNFL thickness may correspond to clinical improvement, particularly in treatment-resistant depression, as seen with repetitive transcranial magnetic stimulation (rTMS) and electroconvulsive therapy [143,144,146]. However, these promising results necessitate further investigation on a larger scale.

## 5. Discussion 

Examining their common linkages reveals that systemic pathophysiological changes, such as inflammation, sleep dysregulation, and metabolic alterations, partly explain the increased prevalence of depression in patients with eye disease [54,108,132]. Notably, iatrogenic causes from pharmacotherapy for either condition contribute to reciprocal pathogenesis [87,92,147,148]. These highlight the importance of understanding the biological contributors to depression and may inform clinicians in opportunistic screening and identification of mental health issues in patients with eye disease. 

Additionally, recognising the retina’s potential as a proxy for vascular health and neurodegeneration in the brain holds diagnostic and prognostic promise [119]. Although its prognostic value remains equivocal, the increasing reliability of approaches to measure parameters such as wall-to-lumen ratios (WLR) of retinal arterioles opens possibilities for non-invasive assessment for microvascular changes [149]. However, further studies will be required to establish and characterise the correlated changes in retinal and ocular examination across various stages of depression. Exploring the reversibility of biological changes in the retina and its alignment with improving depressive symptoms post-treatment necessitates thorough investigation. The prospect of reversing these retinal alterations in tandem with mental health amelioration offers promising avenues for personalised interventions. Integrating mental health interventions into the comprehensive treatment plans for individuals with eye diseases emerges as a pragmatic consideration, addressing immediate ocular concerns and overall well-being.

However, this endeavour encounters challenges, particularly in discerning the extent to which these biological changes contribute to depressive symptoms. Given the multifaceted nature of depression, encompassing biological, psychological, and sociological dimensions, unravelling the specific impact of retinal alterations on depressive symptoms requires nuanced exploration [39]. Addressing these challenges will be pivotal in refining our understanding and optimising the integration of mental health interventions within the broader context of eye disease management.

**Figure 1:**
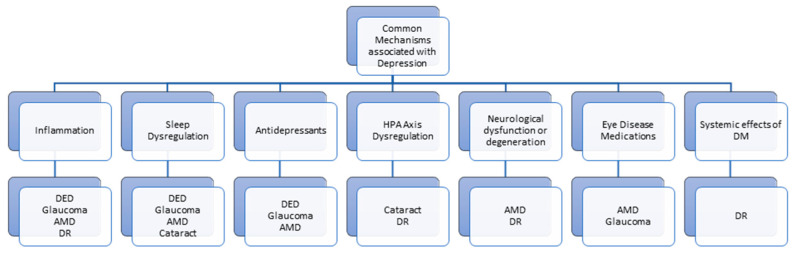
Common mechanisms associated with depression and its correlated eye diseases.

Moreover, the intricate pathophysiology of depression, marked by the absence of a unified hypothesis encompassing all aspects and subtypes, underscores the imperative for further research to delve into these complex mechanisms [39]. Identifying methodologies correlating pathophysiological alterations with clinical measurements is pivotal, potentially enhancing disease monitoring practices. Conducting longitudinal studies, wherein researchers meticulously track patients over extended periods, provides a critical avenue for gaining valuable insights into the temporal dynamics between depressive symptoms and ocular changes. This methodological approach facilitates the discernment of causative relationships and is a foundation for informing proactive and tailored preventive measures (Figure 1).

### Strengths and Limitations

A strength of this narrative review is that it has taken a comprehensive approach to identifying common pathophysiological links between depression and various prominent eye diseases. The investigation highlights shared pathophysiological mechanisms and indicates the potential presence of a retinal–brain–mind axis connection, much like that of the bidirectional relationships noted in the gut–brain axis [150]. The correlation between the retina and the brain could be a promising area for future research. More profound studies in this field could enhance our understanding of the pathophysiology of mental illnesses. This connection opens up exciting possibilities for improving mental health care’s diagnostic, prognostic, and therapeutic aspects.

This study faced challenges due to the inherent heterogeneity in the designs and outcomes of various eye diseases, compromising on the validity of reported associations between depression and ocular disease. Sources of such heterogeneity arise from different patient demographics, varying stages of severities of depression and ocular disease, clinical tools in measuring depressive symptoms, to types of ocular parameters measured. Therefore, the limitations in data harmonisation prevented the study from implementing a quantitative synthesis. Studies may also be prone to selection bias, as depressed patients with anhedonic behaviours may preclude themselves from screening and detection of ocular disease such as diabetic retinopathy [151]. Furthermore, many of the studies reviewed employed a cross-section design in establishing the association between depression and eye disease. Further longitudinal studies elucidating the temporal relationship between depressive symptoms and severity of ocular disease would help to establish the concept of common pathophysiological mechanisms driving both diseases and a probable mind–brain–retinal axis.

In studies assessing the correlation between signs on retinal imaging and depressive symptoms, inconsistencies in findings and limitations to the interpretation of data such as probable interactions between comorbidities and behavioural risk factors with findings from retinal imaging necessitate higher-powered studies across varied populations while controlling for confounding factors [130,131,152]. Although studies assessing OCT parameters show better consistency in results and in suggesting a temporal relationship with severity of depressive symptoms with treatment, these findings may benefit from larger sample sizes with varied populations, as well as correlation with other structural imaging such as MRI [144,145].

The findings of this study provide valuable insights into the relationship between different eye diseases and their potential links to depression. Future studies could adopt more standardised methodologies and outcome measures to enable data synthesis and robust statistical analyses to uncover subtler associations and trends. Understanding their correlation to each other and how their relationship may be measured clinically via non-invasive methods such as retinal imaging or OCT points towards several practical implications. Within the broader context of healthcare, for instance, in ophthalmology practice or in primary healthcare screening of diabetic retinopathy, cases of depression may potentially be identified early and allow for timely intervention. When correlated with functional assessments of depression, these measurements (e.g., RNFL, GCL, IPL) may have a potential role in monitoring and comparing response to treatment such as pharmacotherapy or neurostimulation therapy [143,144,146]. Further conclusive identification of common driving pathophysiological mechanisms between eye disease and depression may also offer novel treatment targets aimed at reversing, monitoring, and preventing ocular pathology in tandem with depression compared to traditional approaches of treating each disease on its own [126].

## 6. Conclusions

In summary, there is substantial evidence of a close association between depression and eye diseases. Both depression and different eye diseases, like DED, cataracts, glaucoma, AMD, and DR, share similar biological mechanisms. The pathophysiology of common mechanisms such as inflammation, sleep dysregulation, and dysregulation of the HPA axis suggests the potential of a probable retina–brain axis. Understanding these concepts can lead to further research on treatment options and monitoring of depression and eye diseases based on ocular parameters.

## Figures and Tables

**Table 1:** Associations between retinal vein occlusion and depression.

Signs on Retinal Imaging	Association with Depression
Retinal venular dilatation	Higher incidence of depressive symptoms [113,116,119]
Retinal arteriole dilatation	Higher incidence of depressive symptoms [116,118]
Plasma markers of endothelial dysfunction	Higher incidence of depressive symptoms [116]
Reduced retinal arterial tortuosity	Increased prevalence of depression [117]
